# A case of facial palsy after spinal surgery with prone positioning in a patient with achondroplasia

**DOI:** 10.1186/s40981-023-00628-6

**Published:** 2023-06-17

**Authors:** Shuto Yoshizawa, Yukio Nomoto, Chiaki Nemoto, Satoki Inoue

**Affiliations:** 1 The Junior Resident Center, Ohara General Hospital, 6-1 Ohomachi, Fukushima, 960-8611 Japan; 2 Department of Otorhinolaryngology, Head and Neck, Ohara General Hospital, 6-1 Ohomachi, Fukushima, 960-8611 Japan; 3 Department of Anesthesiology, Ohara General Hospital, 6-1 Ohomachi, Fukushima, 960-8611 Japan; 4 Department of Anesthesiology, Fukushima Medical University, 1 Hikarigaoka, Fukushima, 960-1295 Japan

**Keywords:** Facial palsy, Prone position, Achondroplasia

To the Editor

The causes of facial palsy vary; however, most cases involve lower motor neurons, and 59–70% are idiopathic, followed by traumatic and viral causes [1]. Facial palsy after prolonged prone positioning during spinal surgery has been reported [2]. Achondroplasia is characterized by skeletal dysplasia, which results in short stature; facial palsy in achondroplasia is reported as a rare association [3].

In the present case, facial palsy was observed after prolonged spinal surgery with prone positioning in a patient with achondroplasia. Even relationship between achondroplasia and traumatic facial palsy is unknown; prolonged compression against prone head positioner might become a risk of traumatic cause of facial palsy during surgery with prone positioning.

A 56-year-old man (height: 138 cm; weight: 53 kg) was scheduled to undergo extensive decompressive laminectomy from Th8 to L5 because of spinal canal stenosis. He had no preoperative comorbidities other than inherited achondroplasia.

General anesthesia was performed with propofol, remifentanil, and rocuronium, with intermittent fentanyl administration. A prone head positioner (Voss Medical Products, San Antonio, TX, USA) was used for patient positioning (Fig. 1). Repositioning of the head was not performed during surgery. The operation duration was 9 h, 16 min; the anesthetic time was 10 h, 45 min. Emergence from anesthesia was smooth; however, we identified pressure sore on patient’s face (Fig. 1). On postoperative day 1, he developed moderate left facial palsy which was evaluated using Yanagihara facial nerve grading system [4]. On postoperative day 8, he was fully recovered.

**Fig. 1:**
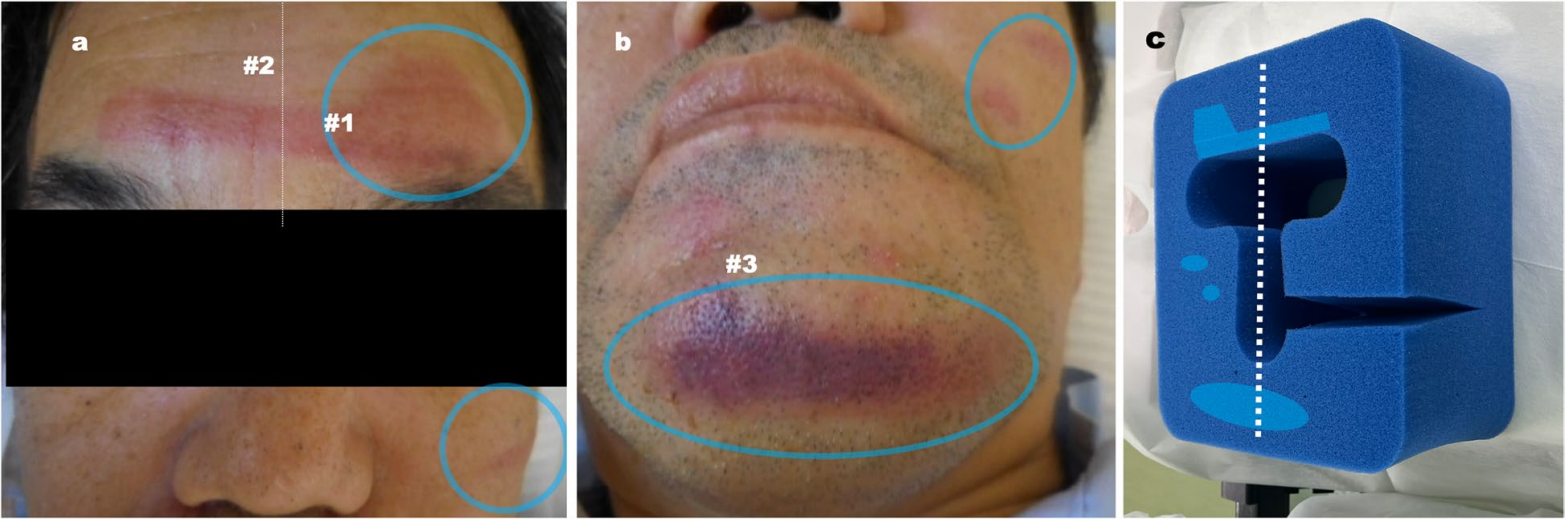
Postoperative findings. Second-grade pressure sores on the forehead, chin, and left cheek (**a** and **b**) and the prone head positioner used during surgery (**c**). **a** and **b** The site of the pressure sores corresponds to craniofacial features of achondroplasia: #1 frontal bossing, #2 short upper facial height, #3 normal-sized prognathic mandible. **c** Light blue indicates the site of contact of the prone head positioner to the pressure sores, predominantly in the left side

Prolonged operation with prone positioning carries the risk of pressure sores, especially on the chest, face, and over the iliac crests. However, facial palsy caused by compression against a facial pillow is less frequent [2]. To protect the patient’s eyes and mouth and to prevent intratracheal tube bending, we used a commercially available prone head positioner. However, although this positioner is soft with polyurethane foam, it is also flat. Flat pillows are associated with relatively high contact pressures compared to other pillows [5]. Moreover, in our case, we tilted the surgical table to the left to secure a good operative field, which also tilted the patient’s face. The resulting pressure sore was markedly left sided, and we considered that the left facial nerve had been stretched and compressed, which led to direct or ischemic damage to the nerve (Fig. 1). Additionally, achondroplasia has marked craniofacial clinical features [3]. It has been suggested that patients with achondroplasia may be sensitive to cranial nerve compression because of inherent craniofacial osseous deformity, which may cause narrowing of the intratemporal nerve course [3]. This characteristic feature might be involved in intraoperative facial nerve compression.

We experienced a patient with achondroplasia and facial palsy that was caused by prolonged prone positioning during spinal surgery. Even though relationship between achondroplasia and traumatic facial nerve palsy is unknown, marked craniofacial clinical features of achondroplasia might exacerbate facial nerve compression during prone positioning. Intraoperatively, relieving facial pressure against the facial pillow may be required at certain intervals.

## Data Availability

Not applicable.
